# Comparison of CLIF-C ACLF Score and MELD Score in Predicting ICU Mortality in Patients with Acute-On-Chronic Liver Failure

**DOI:** 10.7759/cureus.7087

**Published:** 2020-02-24

**Authors:** Moazma Ramzan, Ahtesham Iqbal, Hafiz Ghulam Murtaza, Nasir Javed, Ghulam Rasheed, Khadija Bano

**Affiliations:** 1 Critical Care, Shifa International Hospital, Islamabad, PAK; 2 Internal Medicine, Shifa International Hospital, Islamabad, PAK

**Keywords:** intensive care unit, mortality, clif-c aclf score, meld score

## Abstract

Introduction

Acute-on-chronic liver failure (ACLF) is a serious complication of liver cirrhosis which presents with hepatic and/or extrahepatic organ failure and often needs admission to an Intensive Care Unit (ICU). This condition typically needs organ support and carries a high mortality rate. ICU care may not benefit these patients. There are many scores to assess prognosis in these patients, such as the Model for End-stage Liver Disease (MELD) score, the MELD score refined to take into account serum sodium level (MELD-Na), the chronic liver failure organ failure (CLIF-OF) score, the CLIF Consortium acute-on-chronic liver failure (CLIF-C ACLF) score and the Child-Turcotte-Pugh classification. This study was conducted to compare CLIF-C ACLF and MELD scores for selecting patients at risk of high mortality, as ICU care to these patients in the absence of liver transplantation may be of no value.

Methods

The data of 75 patients admitted to the ICU of Shifa International Hospital in Islamabad were prospectively analyzed. CLIF-C ACLF and MELD scores were calculated at admission and then at 24 and 48 hours after the ICU stay. Data were analyzed with the assistance of SPSS. Mortality was the primary outcome.

Results

Comparison of both scores showed that a CLIF-C ACLF score ≥ 70 at 48 hours predicts mortality more accurately, with an area under receiver operating curve (AUROC) of 0.643 (confidence interval [CI] 95% 0.505-0.781; p=0.046) which was significantly higher than MELD scores of 30,40 and 50 at 48 hours. Organ failure and the need for supportive care were strong predictors of mortality (p= < 0.05).

Conclusion

We concluded that a CLIF-C ACLF score ≥ 70 at 48 hours and organ failure are better predictors of mortality and that ICU care in these patients does not benefit them. Definitive therapy in the form of liver transplantation may have a promising role, if considered early.

## Introduction

Acute-on-chronic liver failure (ACLF) is a serious complication of hepatic cirrhosis, often requiring admission to ICU and organ support. Prognosis depends upon the number and severity of organ failures [1, 2]. Determining prognosis, therefore, helps to monitor treatment response, determine the need for emergency transplantation and provide the rationale for deciding on the futility or otherwise of ICU care.

ACLF is a very serious complication of hepatic cirrhosis that is characterized by hepatic and extrahepatic organ failure and is associated with high short-term mortality [3,4]. Such patients often require artificial support therapies and admission to an intensive care unit. Prognosis of ACLF is related to the number and severity of organ failures [1,2]. Thus, it becomes very important to determine prognosis, as prognosis can help to monitor response to treatment, determine the need for emergency transplantation and provide the rationale for deciding on the futility of ICU care.

The conventional scoring systems, the Model for End-stage Liver Disease (MELD) score, the MELD score refined to take into account serum sodium level (MELD-Na) and the Child-Pugh-Turcotte classification, were designed to predict the prognosis of chronic liver failure. These scoring systems are also commonly used to determine prognosis in ACLF. The accuracy of these scores in predicting the prognosis of ACLF is, however, limited by their inability to incorporate all possible extrahepatic organ failures, which are an important part of the disease spectrum and have a significant impact on prognosis.

Recently, new scoring systems, i.e., the chronic liver failure organ failure (CLIF-OF) score, the CLIF Sequential Organ Failure Assessment (CLIF-SOFA) score and the CLIF Consortium acute-on-chronic liver failure (CLIF-C ACLF) score have been developed and validated to predict short-term mortality in patients with ACLF [5,6]. Of these, the CLIF-C ACLF score has shown better accuracy in predicting mortality [5]. Since ACLF is a dynamic process, serial assessment of the score better predicts mortality and, therefore, can also help in assessing the futility or otherwise of organ support and ICU care

## Materials and methods

Objective

The objective of the study was to compare the CLIF-C CLF score and the MELD score in predicting ICU mortality.

Study design

This was a prospective cohort study.

Sample size

The total sample size was 75, which was calculated by applying Buderer's formula for sample size calculation in sensitivity/specificity analysis, using the following parameters: Expected sensitivity (0.78) [5], Expected specificity (0.74) [5], Expected prevalence (0.88), Desired precision (0.10), and Confidence level (95%).

Diagnosis of ACLF and grading of severity

ACLF was diagnosed using criteria derived from the CLIF OF classification, which is further derived from the CLIF-C SOFA score [3,4]. The CLIF-C ACLF was calculated using the following formula: CLIF-C ACLF = 10 × (0.33 × CLIF- OFs + 0.04 x Age + 0.63 × ln (WBC count)-2) [3]. Organ failure was defined according to Moreau et al. [4]. Grade I ACLF was defined as having acute renal failure or any organ failure along with renal failure; Grade II as having two organ failures; Grade III and Grade IV as ≥ 3 organ failures with a CLIF-C ACLF score ≤ 64 and 4 organ failures with a CLIF-C ACLF score ≤ 64 respectively.

Population and Study Settings

The study was conducted at the Medical ICU/Medical Step Down facilities of the Shifa International Hospital in Islamabad for a period of six months. After approval from the institutional review board (IRB) and ethical committee, informed consent was taken from all participants. Patients of both genders aged > 18 years, admitted to the ICU/Medical Step Down facilities with a diagnosis of ACLF and associated organ failure were included in the study. Those patients whose families asked for withdrawal of intensive care and organ support, who transferred out of hospital < 48 hours after admission to ICU and were < 18 years of age were excluded from the study. Confidentiality was maintained throughout the study. A trainee researcher, who was blind of the objective of the study and adequately trained in filling proforma, recorded demographic and clinical data (serum bilirubin, serum creatinine, international normalized ratio (INR), oxygen pressure in arterial blood (PaO2), peripheral capillary oxygen saturation (SPO2), fraction of inspired oxygen (FiO2), requirement for mechanical ventilation or renal replacement therapy, use of vasopressors, and mean arterial blood pressure) on proforma within one hour of presentation at 24 and 48 hours after admission. The MELD score and the CLIF-C ACLF score were calculated using online calculators [7, 8]. The primary outcome was survival.

Statistical methods:

The collected data were entered and analyzed using the software, Statistical Package for Social Sciences, version 20.0 (SPSS). Descriptive data were expressed as percentages. Statistical analysis was performed to compare the two mortality groups of survivors and non-survivors. Multivariate logistic regression analysis was used to evaluate the risk factors. The receiver operating characteristics (ROC) curve was used to calculate the accuracy, sensitivity and specificity of the two scoring systems. A P-value of <0.05 was considered significant for all tests.

## Results

We studied 75 patients. Of them, 37 (49.3%) were males and 38 (50.7%) were females. Most of the patients were aged between 51 and 60 years (37 patients, 49.3%), and 31 patients were aged between 41 and 50 years (41.3%) (table 1). Our results showed that there is high mortality (66.67%) of ACLF patients with CLIF-C ACLF grade III or above, which is directly proportional to the grade of encephalopathy. Only 25 (33.3 %) patients survived during their stay in ICU. Gender had no statistically significant effect on mortality.

**Table 1:** Age distribution of the study population

Age	Percentage
Age < 41	2%
41-50	41.3%
51-60	49.3%
>61	7%

**Figure 1:**
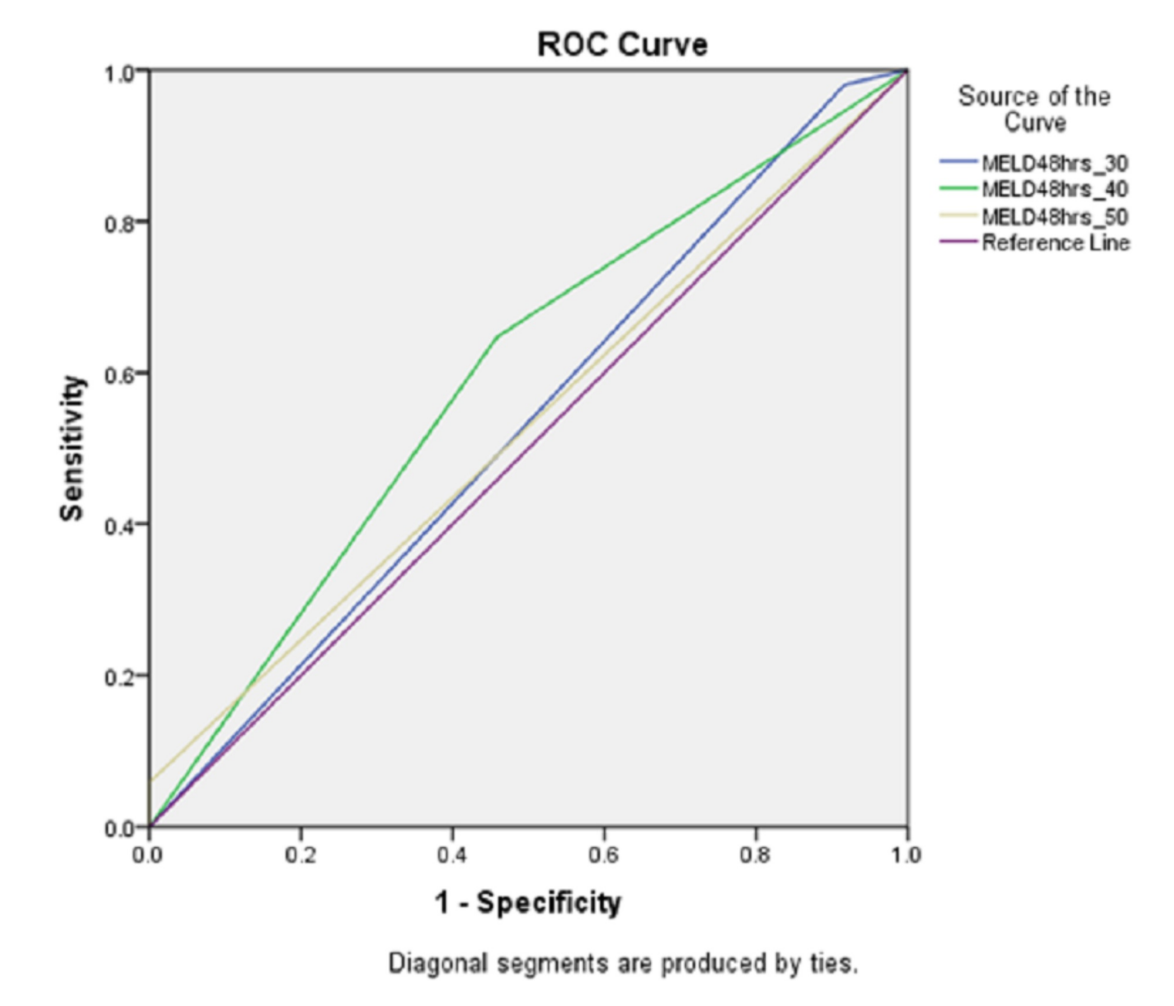
Area under operator receiver characteristic for MELD score 30,40,50 at 48 hours representing specificity

The areas under receiver operating curve (AUROC) for MELD scores 30, 40 and 50 at 48 hours were 0.532 (CI 95% 0.388-0.675; p=0.658), 0.594 (CI 95% 0.455-0.734; p=0.190) and 0.529 (CI 95% 0.391-0.667; p=0.683), respectively (figure 1 and table 2). 

**Table 2:** The test result variable(s): MELD48hrs_30, MELD48hrs_40, MELD48hrs_50 has at least one tie between the positive actual state group and the negative actual state group. a. Under the nonparametric assumption. b. Null hypothesis: true area = 0.5

Area Under the Curve					
Test Result Variable(s)	Area	Std. Error^a^	Asymptotic Sig.^b^	Asymptotic 95% Confidence Interval	
				Lower Bound	Upper Bound
MELD48hrs_30	.532	.073	.658	.388	.675
MELD48hrs_40	.594	.071	.190	.455	.734
MELD48hrs_50	.529	.070	.683	.391	.667

**Figure 2:**
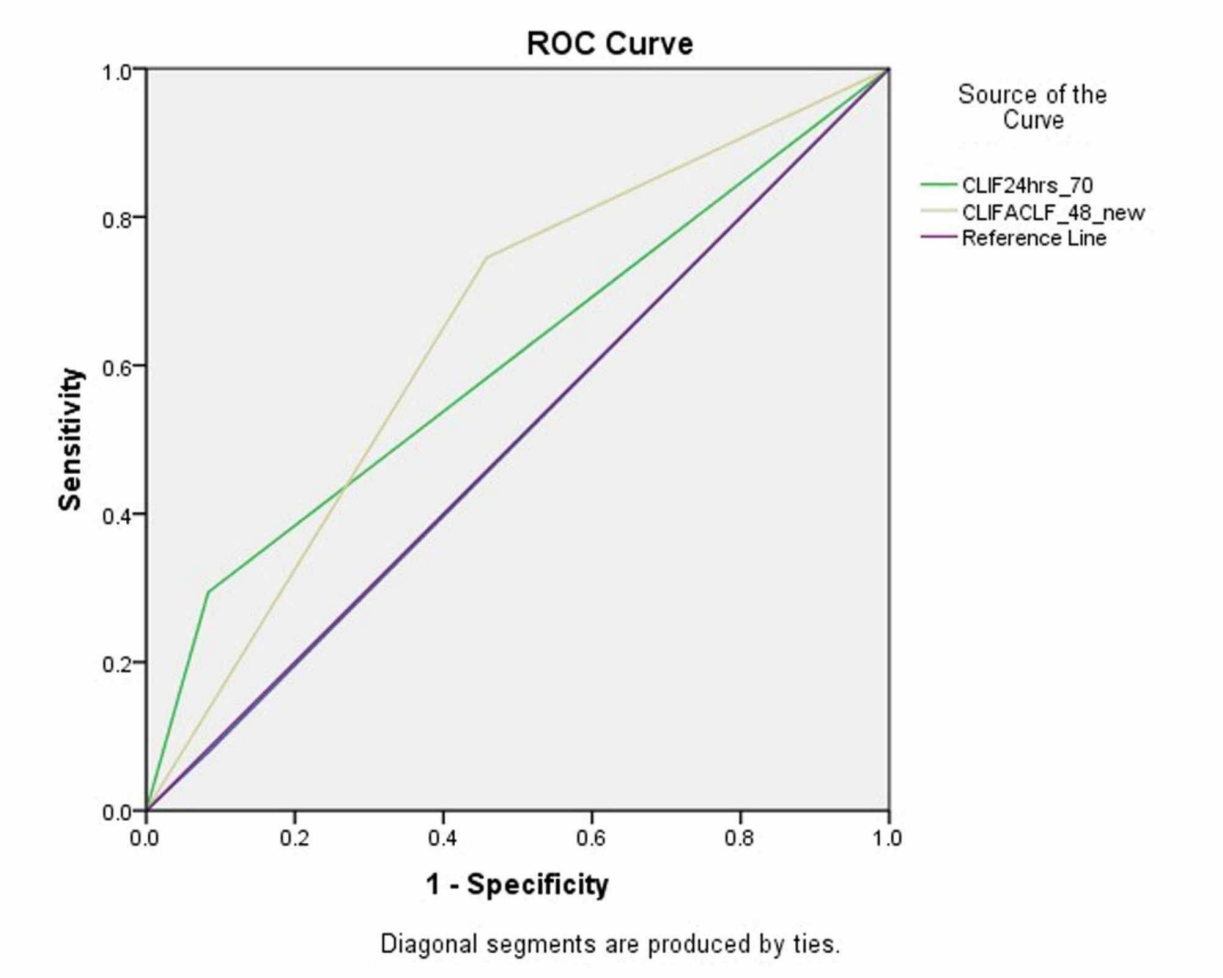
Area under receiver operating curve (AUROC) characteristics for CLIF-C-ACLF at 24 and 48 hours, representing specificity. The cut-off value for CLIF-C-ACLF was 70

The area under receiver operating curve (AUROC) values for a CLIF-C ACLF score ≥ 70 at 0 hours, 24 hours and 48 hours were 0.498 (CI 95% 0.356-0.639 p=0.973), 0.605 (CI 95% 0.474-0.736; p=0.143) and 0.643 (CI 95% 0.505-0.781; p=0.046), respectively (figure 2 and table 3). 

**Table 3:** The test result variable(s): CLIF0hrs_70, CLIF24hrs_70, CLIFACLF_48_new has at least one tie between the positive actual state group and the negative actual state group. Symbol “a” denotes Under the nonparametric assumption whereas “b” stands for Null hypothesis: true area = 0.5

Test Result Variable(s)	Area	Std. Error^a^	Asymptotic Sig.^b^	Asymptotic 95% Confidence Interval	
				Lower Bound	Upper Bound
CLIF0hrs_70	0.498	0.072	0.973	0.356	0.639
CLIF24hrs_70	0.605	0.067	0.143	0.474	0.736
CLIFACLF_48_new	0.643	0.07	0.046	0.505	0.781

The sensitivity and specificity of the CLIF-C ACLF score at 48 hours were 74.51% (60.37% to 85.67%) and 54.17% (32.82% to 74.45%) (CI 95% and p=<0.05), respectively, whereas for MELD, scores of 30, 40 and 50 at 48 hours had 98.04% (89.55% to 99.95%), 64.71% (50.07% to 77.57%) and 5.88% (1.23% to 16.24%) sensitivity (CI 95% and p=<0.05), respectively. Specificity for MELD scores 30, 40 and 50 at 48 hours was 8.33% (1.03% to 27.00%), 54.17% (32.82% to 74.45%), and 100% (85.75% to 100.00%), respectively (CI 95%,p=<0.05) (table 4). 

**Table 4:** Represents sensitivity and specificity of CLIF-C-ACLF at 48 hours and MELD scores of 30, 40 and 50 at 48 hours.

	Sesitivity	(95% CI)	Specificity	Specificity (95% CI)
CLIF 48	74.51%	60.37% to 85.67%	54.17%	32.82% to 74.45%
MELD 30	98.04%	89.55% to 99.95%	8.33%	1.03% to 27.00%
MELD 40	64.71%	50.07% to 77.57%	54.17%	32.82% to 74.45%
MELD 50	5.88%	1.23% to 16.24%	100.00%	85.75% to 100.00%

The accuracy of the CLIF-C ACLF score and the MELD score in predicting survival was assessed by calculating the AUROC. A cutoff value was chosen to accurately predict fatalities with a high specificity. All potential confounders that were part of predictive score calculations for MELD and CLIF-C ACLF were not included in the multivariate model. Patients lost to follow-up were censored at the time of last patient contact. Normally distributed data are presented as mean ±SD and nonparametric data are presented as median (IQR). A P value < 0.05 was considered statistically significant.

Predictors of Mortality

Of the 75 patients, 50 did not survive, with 40 (80%) patients requiring renal replacement therapy within 48 hours of ICU admission (P=0.029). Of the 25 survivors, 21 patients had grade II encephalopathy and four patients had grade III encephalopathy. Amongst the 50 non-survivors, 43 had grade IV encephalopathy (p= <0.001) and seven had grade III encephalopathy. Of the 50 non-survivors, 45 were on vasopressors at 48 hours of ICU stay (p= <0.001). Of the 75 patients, 61 required invasive mechanical ventilation and, of those, 50 patients did not survive while 11 survived (table 5).

**Table 5:** Predictors of mortality

Parameter	Survivors	Non surviovrs	P value
Did not receive renal replacement therapy	(44%)	(20%)	.029
Received renal replacement therapy	(56%)	(80%)	
Mechanical ventilation till 48 hours	(36%)	(98%)	< 0.001
No mechanical ventilation needed at 48 hours	(64%)	(2%)	
Hepatic encephalopathy Grade II	(84%)	(100%)	< 0.001
Hepatic encephalopathy Grade III	(16%)	(14%)	
Hepatic encephalopathy Grade IV	(100%)	(86%)	
On Vasopressors at 48 hours	(08%)	(90%)	< 0.001
No vasopressor at 48 hours	(92%)	(10%)	

## Discussion

Our data shows that ACLF carries a high mortality (%), which is even higher in males compared to females, although this was not found to be statistically significant (p=0.259). A CLIF-C ACLF score ≥ 70 at 48 hours is more accurate (74.51% sensitive and 54.17 % specific) in predicting mortality in ICU than a MELD score at 48 hours. Although maximum treatment was offered to all patients, including optimizing the treatment of comorbidities and providing multidisciplinary care and full organ support, we found that as the MELD score increased, its sensitivity to predict mortality decreased, which challenges its utility in the ICU as a predictor of survival. Our findings are further supported by a study conducted in a tertiary care hospital in Portugal, which involved 289 patients and showed that the CLIF-C ACLF score is a better predictor of mortality than the MELD score (AUROC 0.799, p < 0.05), with a reported specificity of 74% in comparison to our results (specificity 54.17%, sensitivity 74.51% ) [9]. The difference between the two results may be due to our small sample size and the fact that we evaluated our results at 48 hours compared to 28-day mortality, with the CLIF-C ACLF score of 70 as a cut-off compared to 50 in the other study. 

Recently, in 2014, a multicenter study was published in the Journal of Hepatology which showed that a CLIF-C ACLF score ≤ 40 has a 90% negative predictive value and % sensitivity, whereas a CLIF-C ACLF score ≥ 60 has an 82% positive predictive value and 94 % specificity [6]. Although our sample size was small (n=75), 46 patients had an ACLF-C ACLF score ≥ 70 and mortality was (66.67%), implying that patients with a high ACLF-C ACLF score have reduced liver reserves and limited regenerative ability. This may be the reason for the high mortality among these patients, despite receiving maximum treatment for precipitating factors and supportive care to failed organs. In order to reduce mortality in these patients, liver transplantation may be a way forward. The options of early liver transplantation and the risks, benefits and costs of the procedure should be discussed with the family at the time of the patient’s presentation at ICU admission because the time between death and intervention may be very short. Otherwise, ICU care may be a futile effort to treat these patients. We did not follow the survivors (33.33% of total participants) of our study after they were discharged from the critical care unit; hence, mortality beyond this cannot be predicted by our study. However, considering the high mortality within 48 hours of ICU admission, we anticipate that those who survived with high CLIF-C ACLF scores should be evaluated for early liver transplantation in an effort to improve their survival.

According to our above-mentioned results, organ failure and the number of organ failures are key for assessing the prognosis. We found that the need of vasopressors, mechanical ventilation, grade of hepatic encephalopathy and the need for renal replacement therapy were strong predictors of mortality in ICU, in spite of the delivery of best possible care. Liver transplantation in these settings may be a promising option for reducing mortality in these patients.

The MELD score is widely used to predict survival and, is a tool for deciding on candidacy for liver transplant patients. However, our data suggests that an increase in the MELD score is not a good predictor of mortality, raising concerns about whether it should be used among the criteria for liver transplantation. The CLIF-C ACLF score predicts mortality with more sensitivity and specificity at 48 hours, hence, it may be considered a better parameter than the MELD score for selecting patients for liver transplantation in ACLF.

## Conclusions

We concluded that a CLIF-C ACLF score ≥ 70 is a better predictor of ICU mortality in ACLF than the MELD score at 48 hours, and has higher sensitivity and specificity. Organ failures in ACLF are bad prognostic factors and, therefore, the provision of ICU care to these patients may be futile. ICU specialists should involve transplant team and families earlier to discuss the possibility of transplantation and cost of the procedure in order to provide definite treatment option to such patients. Further studies are needed to evaluate the effectiveness of CLIF-C ACLF scores for selecting patients of liver transplantation in ACLF.
